# Complete Genome Sequence of *Staphylococcus lutrae* ATCC 700373, a Potential Pathogen Isolated from Deceased Otters

**DOI:** 10.1128/genomeA.00572-17

**Published:** 2017-06-22

**Authors:** Iva A. Veseli, Chenling Tang, Jean-François Pombert

**Affiliations:** Department of Biology, Illinois Institute of Technology, Chicago, Illinois, USA

## Abstract

Despite their relevance to human health, not all staphylococcal species have been characterized. As such, the potential zoonotic threats posed by uninvestigated species and their contribution to the staphylococcal pangenome are unclear. Here, we report the complete genome sequence of *Staphylococcus lutrae* ATCC 700373, a coagulase-positive species isolated from deceased otters.

## GENOME ANNOUNCEMENT

*Staphylococcus* is a genus of Gram-positive bacteria that can be found colonizing the mucous membranes of animals. They have a wide host range in birds and mammals, including humans, in which they inhabit the skin and oral microbiomes (1). While many staphylococcal species are avirulent, the genus is notorious for including several mild to serious coagulase-positive pathogens, including methicillin-resistant *Staphylococcus aureus* (MRSA) (2). Although much effort has been expended to sequence and assemble the genomes of these medically important species and their relatives, some staphylococci are not yet fully characterized. *Staphylococcus lutrae*, which has been isolated postmortem from deceased otters and may therefore be pathogenic to its host (3), is one such species. To fill this gap in our knowledge and allow for a more comprehensive analysis of the *Staphylococcus* genus, we sequenced and assembled the complete genome of *Staphylococcus lutrae* ATCC 700373.

The *S. lutrae* strain originally isolated by Foster (3) was obtained from the American Type Culture Collection (Manassas, VA, USA). Culturing was done in brain heart infusion (BHI) broth (BD Diagnostics, Franklin Lakes, NJ, USA) as follows. A preculture was prepared by inoculating freeze-dried cells into 7.5 ml of BHI broth and incubating at 37°C for 24 h. A total of 1 ml of preculture was aliquoted into 10 ml of fresh BHI broth, and these cultures were then incubated for 12 h at the same temperature. Genomic DNA was extracted using the MasterPure Gram-positive purification kit (Epicentre, Madison, WI, USA). Library preparation and long-read sequencing were done at the University of Michigan DNA Sequencing Core (Ann Arbor, MI, USA), genomic DNA was sheared with g-TUBEs (Covaris, Woburn, MA, USA), libraries were prepared using the DNA template prep kit 3.0 and the DNA polymerase binding kit P6 version 2 (Pacific Biosciences, Menlo Park, CA, USA), and sequencing was performed on a PacBio RSII sequencer with the C4 chemistry.

Initial assembly of the genome was completed using the HGAP3 protocol in SMRT Analysis version 2.3.0 (4). Another assembly was done in parallel with MHAP, as implemented in Canu version 1.0 (5), using the complete set of subreads from the single-molecule real-time (SMRT) cell. The two assemblies were compared by generating a dot plot with PipMaker (6) and found to match. To circularize the genome, overlapping ends in the longer Canu assembly were trimmed with Artemis version 16.0.0 (7). Base calling was then corrected with the RS_Resequencing.1 protocol in SMRT Analysis. A methylation analysis file was generated with the Modification_and_Motif_Analysis.1 protocol.

The genome (2,533,021 bp; 37.7% G+C; 500× average depth) was annotated using the NCBI Prokaryotic Genome Annotation Pipeline (8).

### Accession number(s).

The whole-genome sequence was deposited in GenBank under accession number CP020773.
